# Risk factors and incidence of postoperative delirium after cardiac surgery in children: a systematic review and meta-analysis

**DOI:** 10.1186/s13052-024-01603-2

**Published:** 2024-02-08

**Authors:** Maoling Fu, Quan Yuan, Qiaoyue Yang, Wenshuai Song, Yaqi Yu, Ying Luo, Xiaoju Xiong, Genzhen Yu

**Affiliations:** 1 Department of Nursing, Tongji Hospital, Tongji Medical College, Huazhong University of Science and Technology, 1095 Jiefang Road, Qiaokou District, Wuhan, Hubei China; 2 School of Nursing, Tongji Medical College, Huazhong University of Science and Technology, Wuhan, Hubei China

**Keywords:** Delirium, Cardiac surgery, Risk factors, Systematic review, Meta-analysis

## Abstract

Delirium, a form of acute cerebral dysfunction, is a common complication of postoperative cardiac surgery in children. It is strongly associated with adverse outcomes, including prolonged hospitalization, increased mortality, and cognitive dysfunction. This study aimed to identify risk factors and incidence of delirium after cardiac surgery in children to facilitate early identification of delirium risk and provide a reference for the implementation of effective prevention and management. A systematic literature search was conducted in PubMed, Web of Science, Embase, Cochrane Library, Scopus, CNKI, Sinomed, and Wanfang for studies published in English or Chinese from the inception of each database to November 2023. The PRISMA guidelines were followed in all phases of this systematic review. The Risk of Bias Assessment for Nonrandomized Studies tool was used to assess methodological quality. A total of twelve studies were included in the analysis, with four studies classified as overall low risk of bias, seven studies as moderate risk of bias, and one study as high risk of bias. The studies reported 39 possible predictors of delirium, categorized into four broad groups: intrinsic and parent-related factors, disease-related factors, surgery and treatment-related factors, and clinical scores and laboratory parameters. By conducting qualitative synthesis and quantitative meta-analysis, we identified two definite factors, four possible factors, and 32 unclear factors related to delirium. Definite risk factors included age and mechanical ventilation duration. Possible factors included developmental delay, cyanotic heart disease, cardiopulmonary bypass time, and pain score. With only a few high-quality studies currently available, well-designed and more extensive prospective studies are still needed to investigate the risk factors affecting delirium and explore delirium prevention strategies in high-risk children.

**Supplementary Information:**

The online version contains supplementary material available at 10.1186/s13052-024-01603-2.

## Introduction

Congenital heart disease (CHD) is the commonest birth defect worldwide, affecting millions of newborns annually [1–3]. The actual prevalence is difficult to determine because not all patients with CHD are diagnosed early. A recent systematic review integrating 260 studies showed that the birth prevalence of CHD globally continued to rise between 1970 and 2017, increasing by 10% every 5 years [4]. With the improvement of medical technology, anesthesia, and extracorporeal circulation, the diagnosis and treatment of CHD have become increasingly sophisticated, and the types of diseases treated have gradually become more complex. Surgery is still the most important and effective treatment for children with CHD, which improves the survival rate of children but may also bring a series of postoperative complications [5]. Delirium is one of the common postoperative complications, with an incidence rate of 9.8%-68% in children undergoing cardiac surgery [6–8]. It is currently receiving increasing attention from the medical community.

Delirium is a manifestation of the acute cerebral dysfunction and is characterized by five features: (1) disturbance in attention and awareness; (2) development over a short period and fluctuating in severity throughout the day; (3) additional disturbances in cognition, such as memory deficit or disorientation; (4) the disturbances in attention, awareness, and cognition not being explained by a known or developing neurocognitive disorder; and (5) history, physical examination, or laboratory findings showing evidence of the disturbances being a direct physiological consequence of one or more etiologies [9]. Despite its typically transient nature, delirium is strongly associated with adverse outcomes, including prolonged hospitalization, increasing potential mortality, impact on prognosis, causing long-term cognitive dysfunction, and differentially affecting the quality of life of the child after discharge from the hospital [10–13]. From an economic perspective, the diagnosis of delirium in children increases medical and healthcare costs [14]. Therefore, prevention has emerged as a pivotal focus of current clinical research.

Identifying risk factors for delirium is crucial for its early identification and prevention, which can effectively decrease its incidence in children. However, there is limited research on delirium after cardiac surgery in children, and the independent risk factors for delirium varied in previous studies. The risk factors and prevalence of delirium have yet to be well established. Therefore, this study aimed to determine the potential risk factors and prevalence of delirium after cardiac surgery in children, to provide a reference for the early clinical identification of high-risk groups, and the implementation of effective prevention and management.

## Methods

The review followed the PRISMA reporting guidelines, a 27-item list designed to improve the reporting of systematic evaluations [15], and was registered with PROSPERO (registration number: CRD42023475618). All relevant analyses were based on previously published studies and did not require ethical approval or patient consent.

### Search strategy

A systematic literature search was conducted in PubMed, Web of Science, Embase, Cochrane Library, Scopus, China National Knowledge Infrastructure (CNKI), Sinomed, and Wanfang for studies that were published in English or Chinese from the inception of each database to November 2023 using keywords, Medical Subject Headings (MeSH), and other index terms, as well as combinations of these terms and appropriate synonyms. The search terms focused on “cardiac surgical procedures,” “thoracic surgical procedures,” “heart surgery,” “cardiac surgery,” “delirium,” “agitation,” “emergence delirium,” “postoperative delirium,” “child,” “pediatrics,” “children,” “neonate,” and their synonyms (see the Supplementary Material for the complete search strategy). Additionally, we manually searched the reference lists of all selected studies for any further relevant studies meeting our inclusion criteria.

### Inclusion criteria

• Pediatric cardiac surgery patients younger than 18 were the primary study population.
• Postoperative delirium is the primary outcome indicator, and risk factors for delirium are the primary research objective.
• Reported at least one statistically significant risk factor for postoperative delirium.
• Published in English or Chinese.

### Exclusion criteria

• Repeatedly published studies.
• Abstracts, clinical trial registries, and medical record reports.
• Conference proceedings, review articles, letters, and editorials.
• Animal or in vitro studies.
• Incomplete data or inability to extract relevant data.
• The original article could not be found by any means.

### Data extraction

Two reviewers extracted the data using a pre-designed Microsoft Excel 2019 spreadsheet. The extraction procedure was conducted independently, and disputes were mediated by a third senior reviewer when necessary. Data were collected on the following characteristics for each included study.

1. Basic information: first author, country, year of publication, study duration, and study design.
2. Demographic characteristics: study population, sample size, number and rate of delirium.
3. Delirium-related features: measurement method and frequency.
4. Potential risk factors for delirium: for each risk factor, adjusted or unadjusted odds ratios (OR) or relative risks (RR) were recorded when available. If not explicitly reported, OR and 95% confidence interval (CI) were calculated with a 2 × 2 table using the number of patients with and without a given risk factor.

### Quality assessment

Two reviewers independently assessed the study’s methodological quality, and disagreements were settled by consensus through a panel discussion. The risk of bias for each included study was assessed using the Risk of Bias Assessment for Nonrandomized Studies tool [16]. This tool was selected because of the nonrandomized nature of all included studies as well as its ability to evaluate six domains of risk of bias, including 1) selection of participants, 2) confounding variables, 3) measurement of exposure, 4) blinding of outcome assessments, 5) incomplete outcome data, and 6) selective outcome reporting. If the study received low risk ratings for each of the six evaluated domains, the risk of bias would be low. If at least one domain were rated to have an unclear risk (but no domains were rated to have a high risk), the study would be at moderate risk of bias, and if at least one domain were rated as having a high risk, the study would be at high risk of bias.

A third reviewer extracted data from five randomly selected studies and examined for methodological quality and bias risk to ensure the correctness of the assessment.

### Qualitative synthesis and quantitative meta-analysis

Each reported risk factor was synthesized qualitatively. The total number of low and moderate risk of bias studies and the percentage of studies showing positive correlation were used to mark them as definite, likely, unclear, or not a risk factor (Table 1). For risk factors with sufficiently homogeneous definitions and reference ranges, a quantitative meta-analysis of low and moderate risk of bias studies was implemented to estimate a combined OR.

**Table 1:** Defining the strength of a risk factor

**Definite**
All low and moderate risk of bias studies positive (at least three studies)
Majority (more than 50%) low and moderate risk of bias studies positive (at least five studies)
**Likely**
All low and moderate risk of bias studies positive (two studies)
Majority (more than 50%) low and moderate risk of bias studies positive (2–4 studies)
**Unclear**
All low and moderate risk of bias studies positive (one study)
Low and moderate risk of bias studies show mixed or conflicting results
A majority (more than 50%) of studies negative but at least one low or moderate risk of bias study positive
**Not a risk factor**
No low or moderate risk of bias studies positive

Data analysis was performed using Revman5.4 software provided by the Cochrane Collaboration Network. The generic inverse variance method was used for the meta-analysis of both risk factors and the incidence of delirium after cardiac surgery in children [17]. This method requires only effect estimates and their Standard Errors (SEs). The SEs were estimated by back transforming the 95% confidence limits using the standard normal distribution. The included studies were tested for heterogeneity (I^2^ test), if *P* ≥ 0.05 and I^2^ < 50%, this indicated less heterogeneity among studies and a fixed-effects model was selected for statistical analysis of the data, while conversely *P* < 0.05 or I^2^ ≥ 50% indicated greater heterogeneity among studies and a random-effects model was used.

## Results

**Fig. 1:**
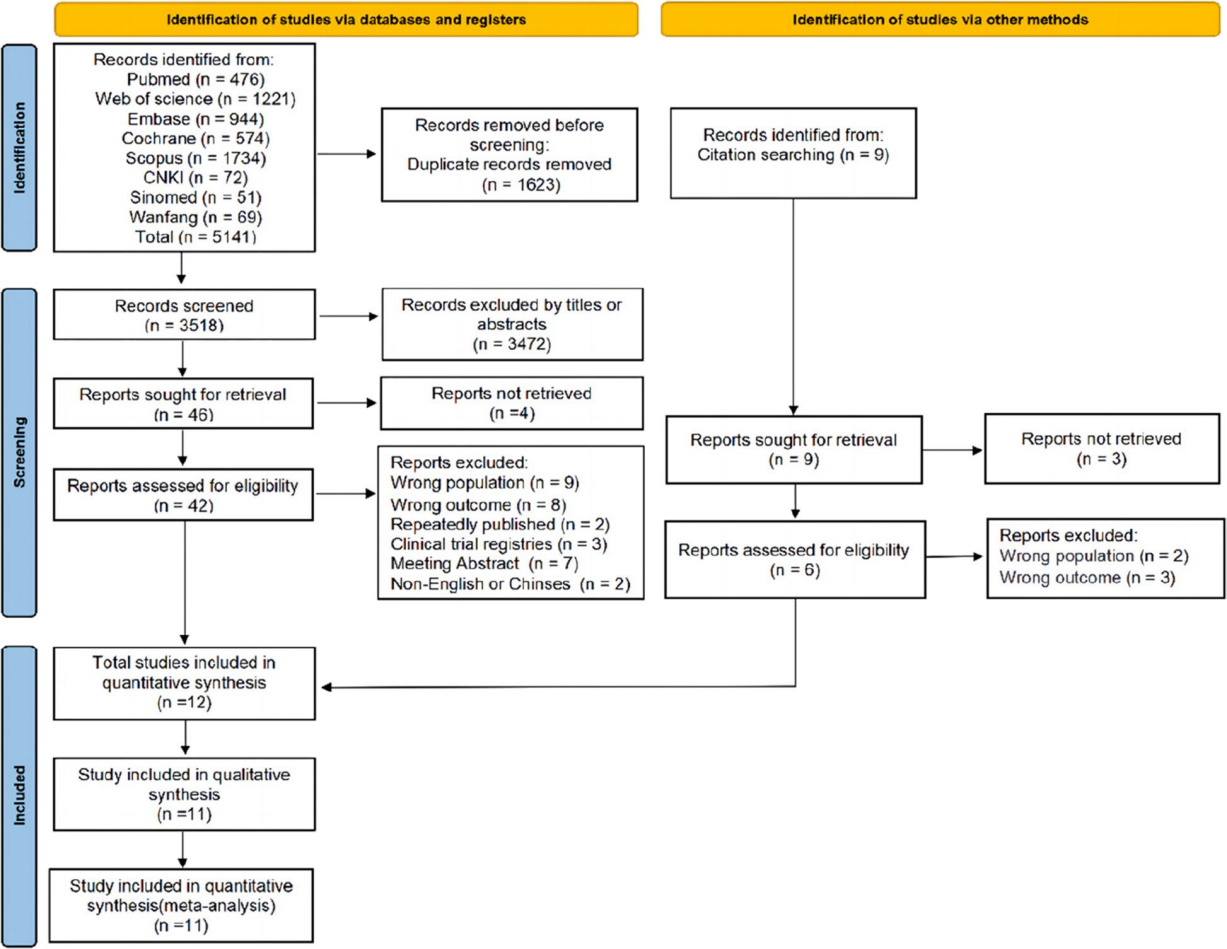
Flow chart of the systematic literature search

A total of 5141 articles were identified through a literature search of the databases, of which 1623 duplicates were removed. After screening the remaining 3518 articles for title and abstract, 46 were selected for full-text retrieval. Following the eligibility assessment, 11 articles were found to meet the inclusion criteria. The references of the selected articles were also checked, and a full-text search was performed on nine articles, including one article that met the eligibility criteria. Ultimately, 12 articles were identified for article inclusion in this review, with 11 studies contributing to qualitative synthesis and quantitative meta-analysis. Study identification is summarized in Fig. 1.

Of these studies, nine were prospective studies [6–8, 10, 18–22], and the remaining three studies were retrospective [23–25], with one being a multicenter studies [8] and 11 being single-center studies. The sample size ranged from 18 to 470, with the two largest studies including 470 children [20] and 327 children [7], respectively. One of these studies constructed clinical prediction models [20]. The basic characteristics of the included literature are given in Table 1.

### Incidence of delirium and measurement method

The combined incidence of delirium was 39.0% (95% CI: 28.6–50.2%). The heterogeneity was high (I^2^ = 95%). The combined incidence of delirium was 40.8% (95% CI: 27.0–56.9%; I^2^ = 92%) in female children and 45.1% (95% CI: 29.6–61.2%; I^2^ = 94%) in male children. (Fig. S1-3 in the Supplementary material).

Two studies used the Pediatric Anesthesia Emergence Delirium (PAED) scale [26] to assess delirium in children. The scale was developed by Sikich et al. in 2004 includes five items: (1) The child makes eye contact with the caregiver, (2) the child’s actions are purposeful, (3) the child is aware of his/her surroundings, (4) the child is restless, and (5) the child is inconsolable. Items 1, 2, and 3 are reverse-scored as follows: 4, not at all; 3, just a little; 2, quite a bit; and 1, very much; 0, extremely. Items 4 and 5 are scored as follows: 0, not at all; 1, just a little; 2 quite a bit; 3, very much; and 4, extremely. A total PAED scale score was calculated by summing the scores of each item, with higher scores indicating more severe delirium. Children with a PAED score ≥ 10 were considered to have emergence delirium.

Ten studies assessed delirium in children using the Cornell Assessment of Pediatric Delirium (CAPD) scale, an instrument developed by Silver et al. for assessing delirium in patients aged 0–21 years [27]. The CAPD scale has eight items: (1) eye contact with the caregiver, (2) purposeful actions, (3) awareness of surroundings, (4) ability to communicate needs and wants, (5) restlessness, (6) difficulty calming, (7) underactive, and (8) long time to respond to interactions. Each item is scored on a scale of 0 to 4, with the fewer symptoms and behaviors from items 1–4, the higher the score. The more symptoms and behaviors from items 5–8, the higher the score. A child with a total score of ≥ 9 points is diagnosed with delirium.

### Quality of included studies

**Fig. 2:**
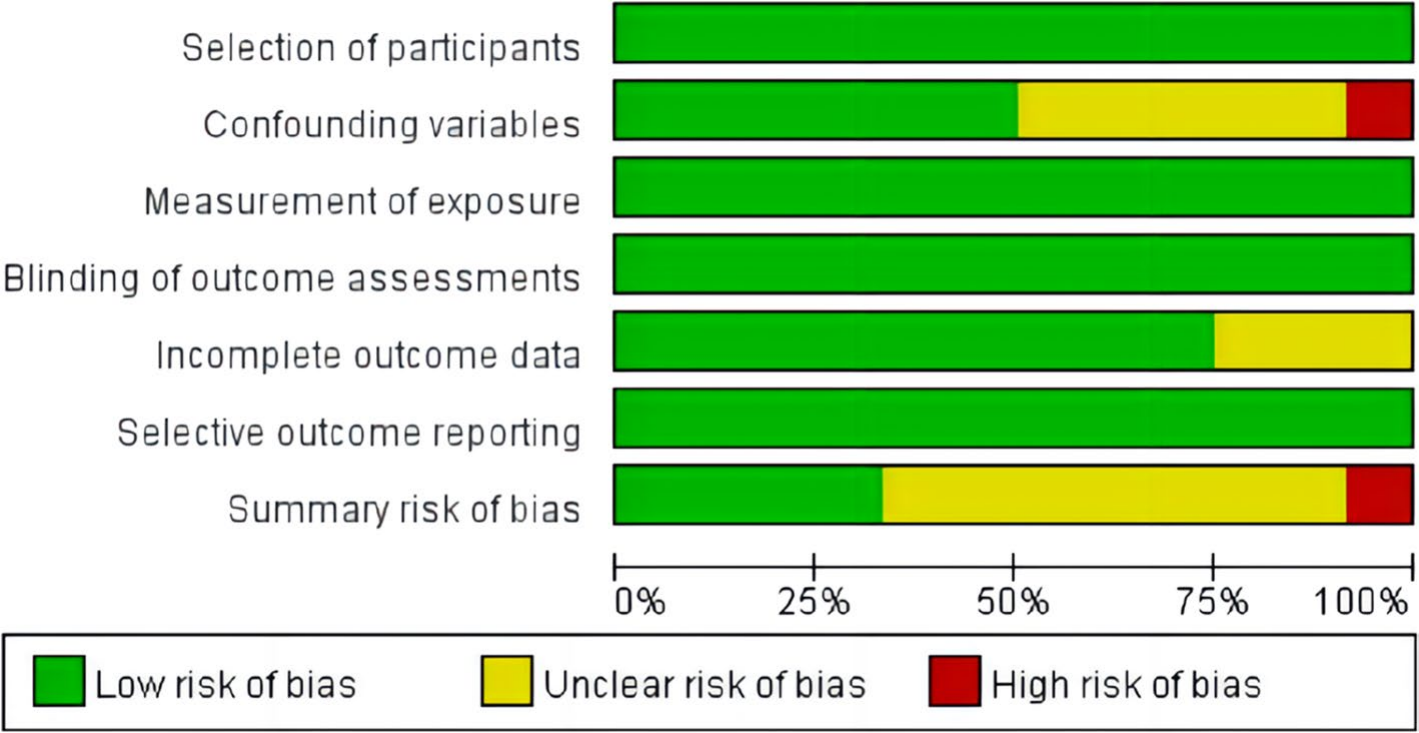
Summary of risk of bias in the included studies

The included studies differed in their methodological quality (Fig. 2, and Fig. S4 in the Supplementary material). Four studies were classified as low risk in all six domains and were considered to be at an overall low risk of bias. One study was considered to be at overall high risk of bias, associated with confounding variables. The remaining seven studies had at least one unclear risk in six domains and were categorized as having a moderate risk of bias.

### Risk factors of delirium in included studies

The 12 included studies describe 39 statistically significant risk factors for delirium. These variables were divided into four major categories: intrinsic and parent-related factors (12.8%, 5/39); disease-related factors (18.0%, 7/39); surgery and treatment-related factors (48.7, 19/39) and clinical scores and laboratory parameters (20.5%, 8/39). Details of the risk factors identified in each study are presented in Table 2.

**Table 2:** Summary characteristics of the 12 studies included in this review

Author	Country of study	Year of publication (data)	Study Design	Sample sources	Study population	Sample size	Delirium rate	Delirium measurement	Frequency	Potential risk factors	Study quality
Alvarez	USA	2018 (2015)	prospective cohort	single-center	Children between 0 and 21 years old	88	52 (59.1%)	CAPD score	every 12 h	gender, age, cyanotic heart disease, STS-EACTS score, CPB time	moderate
Gregory	USA	2021 (2016–2017)	prospective cohort	single-center	children ≥ 6 months of age with cardiac bypass surgery	18	11 (61.1%)	CAPD score	every 12 h	sleep disruption	moderate
Köditz	Germany	2023 (2019–2021)	prospective cohort	single-center	Children between 0 and 18 years	89	61 (68.5%)	CAPD score	after the ventilation phase	depth of anesthesia, age	moderate
Li	China	2015 (2011–2013)	retrospective cohort	single-center	Children < 36 months of age and diagnosed with PAH	174	45 (25.9%)	PAED scale score	When the child awakens or becomes agitated	dexmedetomidine	moderate
Liu	China	2023 (2020–2022)	retrospective cohort	single-center	Children < 1 year old, and rScO2 desaturation of 10% from baseline for more than 30 s during surgery	61	24 (39.3%)	CAPD score	every 12 h	aortic cross-clamp time, mechanical ventilation duration, severity of intraoperative rScO2 desaturation	moderate
Lyu	China	2022 (2020–2021)	prospective cohort	single-center	Children between 0 and 7 years	216	114 (52.8%)	CAPD score	every 12 h	age, gender, number of invasive catheters per day, pain score, preoperative parental anxiety level, developmental delay	low
Mao	China	2023 (2021)	prospective cohort	single-center	Children between 0 and 8 years	470	120 (25.5%)	CAPD score	8:00, 14:00, and 20:00 every day and at times of abnormal situations	age, (PRISM) III, non-invasive ventilation after extubation, delayed chest closure, phenobarbital dosage, promethazine dosage, mannitol usage, elevated temperature	low
Matsuishi	Japan	2022 (2019–2020)	prospective cohort	single-center	Children between 0 and 18 years old	40	17 (42.5%)	CAPD score	up-to3-day post-operative (up to 4 days total)	brain injury marker levels: NSE	moderate
Patel	USA	2017 (2014–2015)	prospective cohort	single-center	Children ≤ 21 years old with cardiac bypass surgery	194	95 (49.0%)	CAPD score	at least once a day	age, developmental delay, RACHS-1 score, cyanotic heart disease, albumin level	low
Staveski	USA	2021 (Not mention)	prospective cohort	multicenter	Children between 0 and 18 years old	181	73 (40.3%)	CAPD score	at 06:00 local time	pain score, total opioid exposure, SBS less than 0, pain medication or sedative administered in the previous 4 h, no progressive physical therapy or ambulation schedule, parents not at bedside at time of data collection, EMCO, mechanical ventilation duration, vasoactive agents, number of invasive catheters	moderate
Xu	China	2020 (2019)	retrospective cohort	single-center	children receiving transthoracic device closure of VSD	68	13 (19.1%)	PAED scale score	Not mention	sufentanil	high
Yang	China	2021 (2016–2020)	prospective cohort	single-center	Children ≤ 15 years old with CPB	327	32 (9.8%)	CAPD score	every 12 h	age, CPB time, aortic cross-clamp time, type of cardiac malformation, length of ICU stay, mechanical ventilation duration, preoperative pulmonary infection, ventilator complications, postoperative complications	low

Two variables were found to be definite risk factors for delirium, based on either all low and moderate risk of bias studies showing a positive association (if at least three studies) or the majority of low and moderate risk of bias studies showing a positive association (if at least five studies). Definite risk factors included age and mechanical ventilation duration. Four variables were considered likely associated with delirium, and these included being of developmental delay, cyanotic heart disease, cardiopulmonary bypass (CPB) time, and pain score. 32 variables that showed conflicting results in studies with low and moderate risk of bias, or were positive in only one study, that were considered to have an unclear association with delirium (see Table 2 for specific variables). In addition, sufentanil was considered a non-risk factor.

**Fig. 3:**
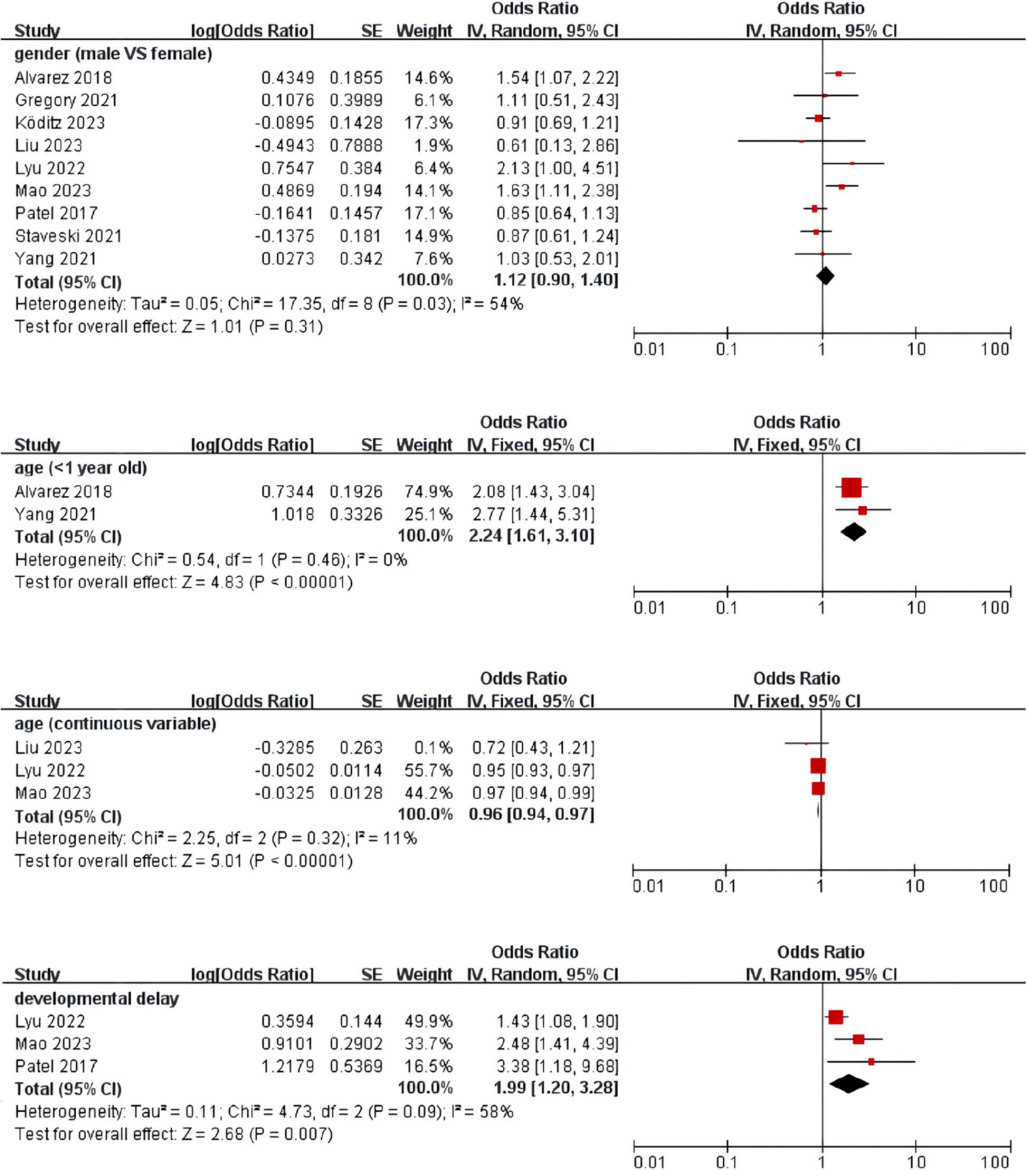
Meta-analysis of intrinsic factors. Forest plots of odds ratios (ORs) that were included in the quantitative meta-analysis and the associated overall ORs. For each OR, the size of the red square region is proportional to the corresponding study weight. Diamond shapes intervals represent the overall ORs. I^2^ represents the fraction of variability among the individual ORs that cannot be explained by sampling variability

**Fig. 4:**
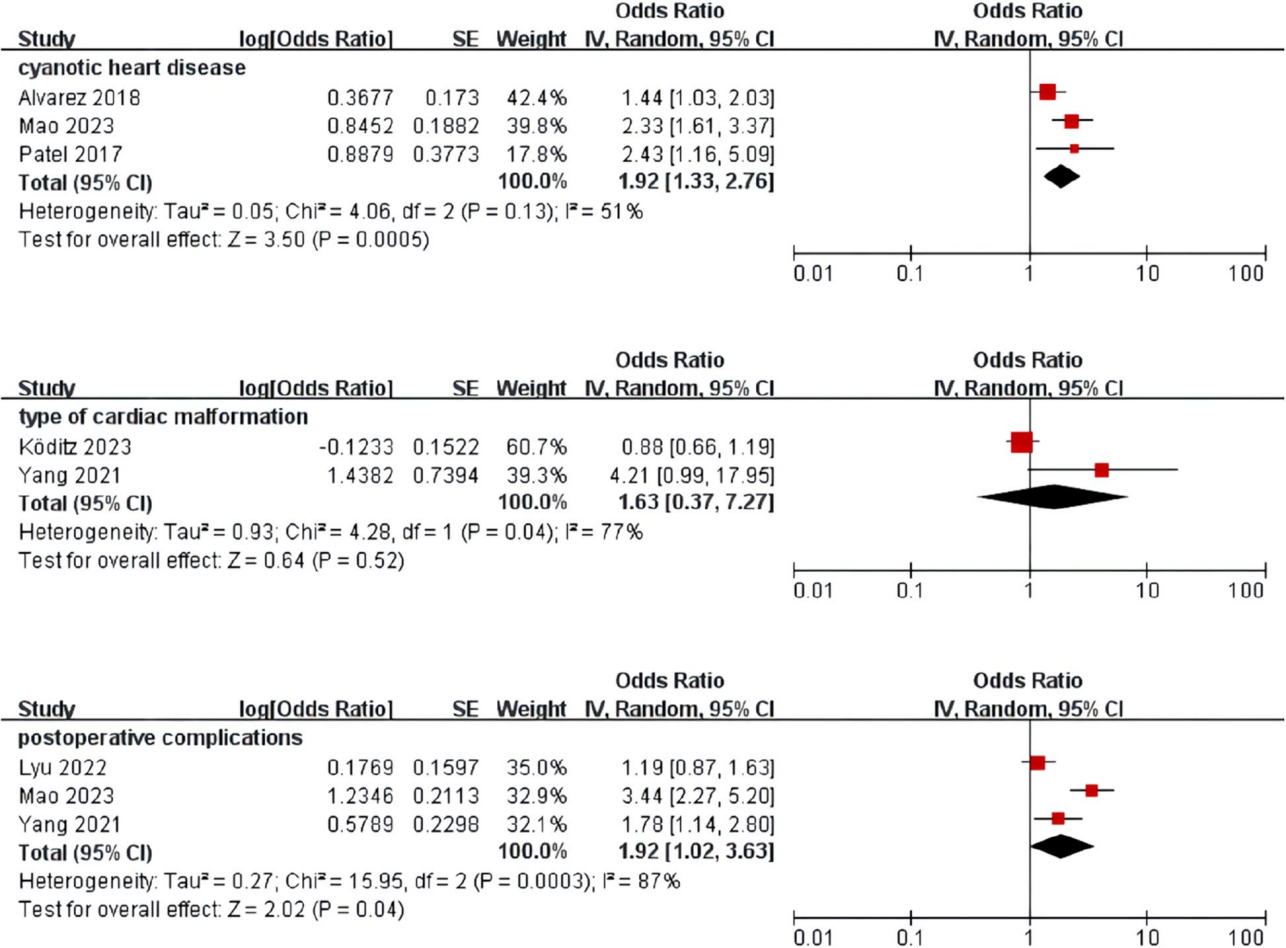
Meta-analysis of disease-related factors

**Fig. 5:**
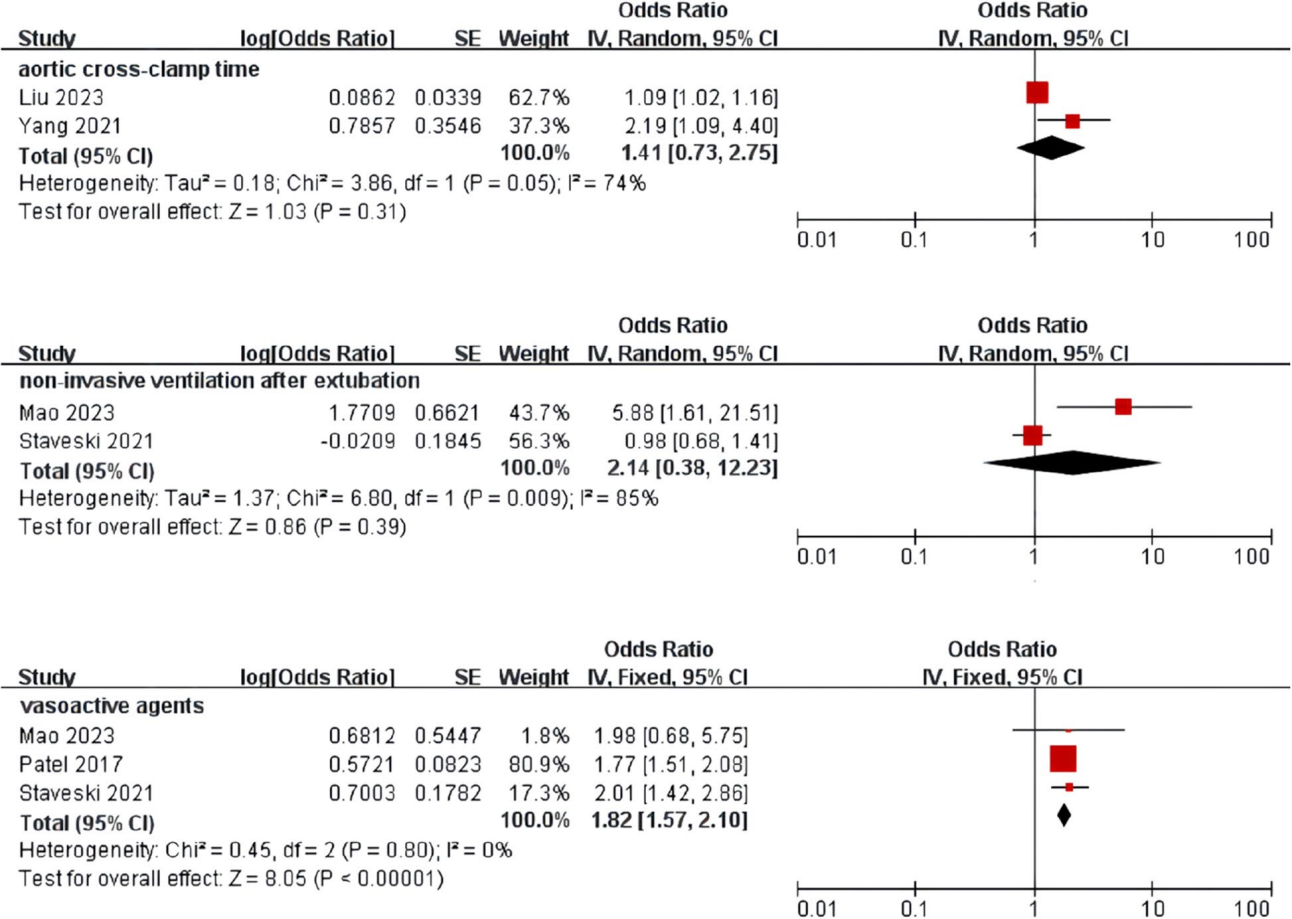
Meta-analysis of surgery and treatment-related factors

**Fig. 6:**
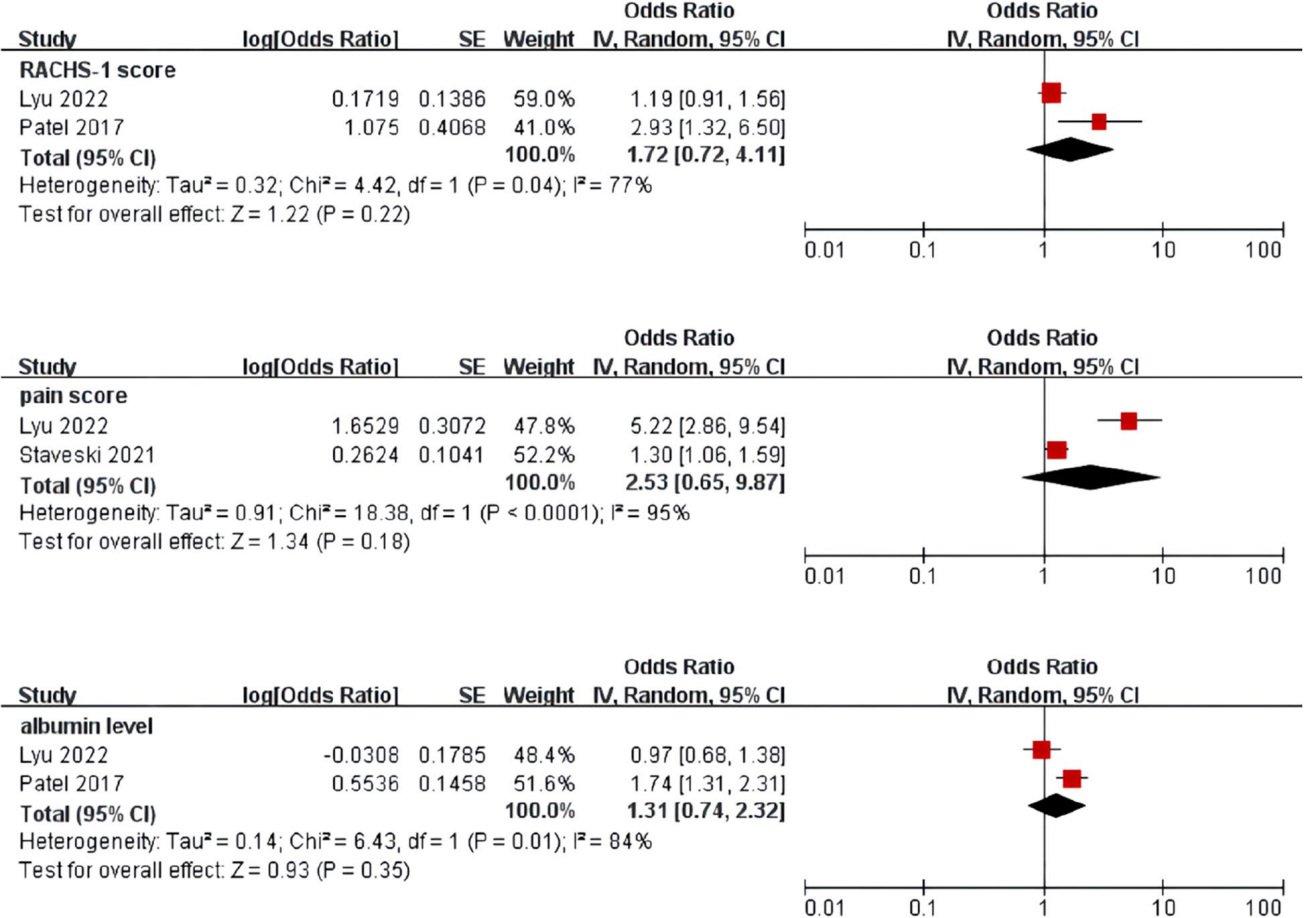
Meta-analysis of clinical scores and laboratory parameters

Meta-analysis was performed for risk factors with at least two low or moderate risk of bias studies demonstrating homogeneous risk factor definitions and reference ranges (Figs. 3, 4, 5 and 6).

## Discussion

Our study was the first systematic review of risk factors for delirium after cardiac surgery in children. Based on the inclusion of 12 studies in the current meta-analysis, involving a total of 1976 patients, the pooled prevalence of delirium is 39.0%. By conducting a qualitative synthesis of 39 predictors and a quantitative meta-analysis of 13 factors, we identified two definite factors, four possible factors, and 32 unclear factors related to delirium. The definite factors included age and mechanical ventilation duration, while the possible factors included developmental delay, cyanotic heart disease, CPB time, and pain score. The results of our systematic review present an up-to-date comprehensive summary of the latest evidence, which can provide information for early identification of high-risk delirium after pediatric cardiac surgery and the development of interventions to reduce and prevent delirium.

The pathophysiology of delirium is not fully understood, but several theories have been put forth to explain the neuropsychiatric disturbances. Possible etiologic factors include brain changes revealed by neuroimaging, sepsis-related inflammation, genetics, biomarkers, and neurotransmitters [28–30]. The incidence of delirium in children after cardiac surgery is generally higher than in the group of nonsurgical children in the intensive care unit, which may be attributed to the correlation between the severity of the disease and delirium in children [31]. In comparison to nonsurgical critically ill children, children who undergo cardiac surgery exhibit more pronounced characteristics of critical illness, including specific risks such as preoperative hypoxemia, neurodevelopmental abnormalities, fluid overload, electrolyte disorders, and hypothermia [32]. Additionally, the extracorporeal circulation techniques commonly employed in pediatric cardiac surgery may induce extensive endothelial cell activation, systemic inflammatory response, and thromboembolic events [33], which in turn lead to brain damage and inflammation, hence the higher incidence of delirium in children after cardiac surgery [10, 22, 34]. Delirium can lead to prolongation of hospitalization, increased healthcare costs, impaired cognitive function, and higher mortality, causing immediate and long-term harm to patients. Hence, early identification of delirium helps healthcare professionals to take preventive and therapeutic measures as early as possible to reduce the adverse effects of delirium.

Our findings align with previous research indicating that age is the most critical risk factor affecting delirium [6, 7, 10, 19, 20, 22]. The younger the age, the higher the likelihood of delirium. Maldonado proposed a related neuropathological hypothesis known as neuronal aging [35], whereby changes in intracellular signaling, stress-regulated neurotransmitters, and cerebral blood flow all lead to neuronal loss. The hypothesis may have a counterpart at the other extreme of age, a fragile immaturity hypothesis. Considering the infant brain, continuous central nervous system (CNS) regional development, neurogenesis, migration, synaptogenesis, and myelination reflect the immaturity of the CNS. These developing brains may exhibit heightened vulnerability to delirium due to the impact of stress and disease. Further research is still needed to explore the pathophysiology of delirium in young children and the impact of delirium on their long-term neuropsychiatric health.

Developmental delay was identified as a possible risk factor for delirium. Children with developmental delay in the CNS are more susceptible to the effects of cardiac bypass, anesthesia, and surgery [22]. Furthermore, due to the challenge faced by evaluators in determining whether the level of consciousness and cognition of children with developmental delays is altered compared to their baseline conditions, more precise tools are needed to assess the interaction between developmental delay and delirium. Lyu et al. [19]. demonstrated that the risk of delirium in male children was 2.127 times higher than in female children. Similarly, a study by Alvarez et al. [10] indicated a higher probability of delirium in male children. However, other studies have not found a significant association between gender and the occurrence of delirium [6, 8, 18, 22, 24]. Moreover, the European Society of Anesthesiology evidence-based and consensus guidelines on postoperative delirium do not recommend gender as a risk factor for delirium [36]. Therefore, further research is warranted to expand the scope of studies and explore the relationship between gender and delirium.

Diseases and adverse conditions are not only the major cause of prolonged hospitalization and even death in children but may also be important risk factors for delirium. The increased risk of delirium in children with cyanotic heart disease may be due to the effects of prolonged chronic hypoxia, oxidative stress, blood transfusions, and poor nutritional status, all of which may independently act as predisposing factors for delirium [37, 38]. The majority of patients admitted to the pediatric cardiac intensive care unit (PCICU) after cardiac surgery are under 3 years of age [39], an age when neurocognitive development is rapid and healthy sleep is critical. Sleep disturbances are recognized as a potential risk factor for the development of delirium, and children with delirium frequently exhibit disrupted sleep patterns. Data from a study by Gregory et al. suggested that the majority of children in the PCICU had severe sleep disturbances [18]. It is necessary to further explore the importance of improving postoperative sleep status in children undergoing cardiac surgery, as well as the feasibility of continuous dynamic monitoring of sleep in the ICU environment. In addition, one study showed that preoperative lung infections and postoperative complications were risk factors for delirium [7], with the possible explanation of these exacerbating the severity of the disease. The more severe the patient's condition, the higher the risk of postoperative delirium [40]. Therefore, it is important to promptly treat preoperative infections and minimize the risk of complications, as this may help prevent delirium.

CPB is a form of assisted circulation specific to cardiac surgery, but this process is non-physiological. Non-pulsatile blood flow affects cerebrovascular autoregulation and interferes with matching cerebral blood flow to metabolism. Hypothermia during CPB is unfavorable for oxygen release. During the warming period, the metabolism of the brain accelerates and the demand for oxygen in the brain increases [41, 42]. Alvarez's study found that children with delirium had longer CPB durations [10], which aligns with the findings of Yang et al. [7]. In addition, pediatric cardiac surgery patients often require postoperative mechanical ventilation, prolonged analgesia, and sedation to improve the safety of tracheal intubation. However, prolonged and continuous mechanical ventilation increases the risk of low cardiac output syndrome, respiratory failure, and severe insufficiency of cerebral blood supply in children [43], resulting in a greater susceptibility to delirium. This suggests that we should make adequate preoperative preparations for children undergoing CPB to minimize the CPB time, the duration of mechanical ventilation, and the occurrence of various complications.

Accurate selection and evaluation of the appropriate dosage of sedative and analgesic medications are particularly crucial in children at high risk for delirium [42]. A planned sedation and analgesia regimen can reduce the likelihood of delirium to some extent. In recent years, dexmedetomidine (Dex) has been increasingly used in children [23, 44, 45]. Dex, a selective alpha-2-adrenergic receptor agonist with sedative, anxiolytic, and analgesic effects without causing significant respiratory depression, has been recommended by the Society of Critical Care Medicine as the primary sedative for critically ill pediatric postoperative cardiac surgical patients [46]. A growing body of research supports that Dex is less likely to induce delirium compared to benzodiazepines [23, 47–49]. However, intravenous infusion of Dex may lead to dose-dependent hypotension and bradycardia due to its potential sympathetic activity [50]. In addition, given the limited high-quality evidence available, there is an urgent need for more high-quality randomized controlled trials (RCTs) to further define the short- and long-term safety and feasibility of Dex for pediatric cardiac surgery.

Pain score is recognized as a possible risk factor. Lyu et al.’s study demonstrated that the risk of delirium in children with moderate to severe postoperative pain was 5.856 times higher than that of children with no pain and mild pain [19]. Previous studies have also indicated that relieving postoperative pain can effectively prevent and treat delirium during postoperative awakening in pediatric patients [51, 52]. The mechanism remains unclear and may be related to the fact that the pain-induced stress response produces persistently high levels of cortisol, which impairs the function of the central nervous system and thus causes delirium [53]. Disease severity also directly affects the incidence of delirium and serves as an independent risk factor [12, 54–56]. There are many measures of disease severity in children, and commonly used ones include the Risk Adjustment in Congenital Heart Surgery-1 (RACHS-1) score, the Society of Thoracic Surgeons and the European Association for Cardiothoracic Surgery Congenital Heart Surgery Mortality (STS-EACTS) Score and Pediatric Risk of Mortality (PRISM) III. In the study by Patal et al., children with a RACHS-1 score of 2 accounted for 40% of the total number of children with delirium and were usually associated with multiorgan dysfunction [22]. Similarly, Mao et al. utilized (PRISM) III to assess the severity of a child's condition and observed a positive correlation between higher scores and an increased likelihood of developing delirium [20].

The strengths of this systematic review include the systematic approach to identifying all publications containing risk factors for delirium after pediatric cardiac surgery and the division of risk factors into four major categories to provide a logical progression of possible factors of delirium. Nevertheless, the results of this systematic review and meta-analysis must take into account several limitations. First, the vast majority of studies were single-center studies, and most were conducted in the United States and China, which may limit their generalizability. Second, there is a lack of standardization in screening for delirium, and the CAPD is usually done by day shift nurses. Consequently, children with nighttime-only delirium may go undetected, which may lead to an underestimate of the true incidence and duration of delirium. Furthermore, despite the comprehensive and rigorous search strategy employed, it is possible that some studies may have been inadvertently overlooked. Lastly, and most importantly, our pooled analyses describe associations between specific factors and the odds of developing delirium, but these observations do not establish causality.

## Conclusions

In summary, we identified several of the most critical factors affecting delirium in our published studies, including age, mechanical ventilation duration, developmental delay, cyanotic heart disease, CPB time, and pain score. However, none of the included studies considered the impact of sociodemographic factors (e.g., family income), skill level of the intensive care unit team, and level of environmental management (e.g., light and sound exposure) on delirium. Therefore, future research should focus on well-designed and extensive prospective studies to investigate the risk factors associated with delirium and explore prevention strategies for high-risk children.

### Supplementary Information

**Additional file 1.**

## Data Availability

The datasets used and/or analysed during the current study are available from the corresponding author on reasonable request.

## Abbreviations

CHD: Congenital heart disease
CNKI: China National Knowledge Infrastructure
OR: Odds ratios
RR: Relative risks
CI: Confidence interval
SEs: Standard Errors
I^2^: Heterogeneity
PAED: Pediatric Anesthesia Emergence Delirium
CAPD: Cornell Assessment of Pediatric Delirium
CPB: Cardiopulmonary bypass
CNS: Central nervous system
PCICU: Pediatric cardiac intensive care unit
Dex: Dexmedetomidine
RACHS-1 score: Risk Adjustment in Congenital Heart Surgery-1 score
STS-EACTS: Society of Thoracic Surgeons and the European Association for Cardiothoracic Surgery Congenital Heart Surgery Mortality Score
(PRISM) III: Pediatric Risk of Mortality III
PAH: Pulmonary arterial hypertension
rScO2: Regional cerebral oxygen saturation
NSE: Neuron-specific enolase
SBS: State Behavioral Scale scores
EMCO: Extracorporeal membrane oxygenation
VSD: Ventricular septal defect
