# Supplementary material for: Risk factors and incidence of postoperative delirium after cardiac surgery in children: a systematic review and meta-analysis
Source: Ital J Pediatr. 2024 Feb 8;50:24. doi: 10.1186/s13052-024-01603-2 (PMC10854157; doi:10.1186/s13052-024-01603-2)
Supplement: Supplementary file 1 — Additional file 1. [file 13052_2024_1603_MOESM1_ESM.docx]

**Supplemental Digital: Database search strings**

**Pubmed**

(((((("Cardiac Surgical Procedures"[Mesh]) OR ("Thoracic Surgical Procedures"[Mesh])) OR ("Thoracic Surgery"[Mesh])) OR ((((((((((((((((((("cardiac surgical procedures"[Title/Abstract]) OR ("cardiac surgical procedure"[Title/Abstract])) OR ("thoracic surgical procedures"[Title/Abstract])) OR ("thoracic surgical procedure"[Title/Abstract])) OR ("thoracic surgery"[Title/Abstract])) OR ("heart surgical procedure"[Title/Abstract])) OR ("heart surgical procedures"[Title/Abstract])) OR ("cardiac valve annuloplasty"[Title/Abstract])) OR ("mitral valve annuloplasty"[Title/Abstract])) OR ("heart massage"[Title/Abstract])) OR ("heart transplantation"[Title/Abstract])) OR ("heart-lung transplantation"[Title/Abstract])) OR ("heart valve prosthesis implantation"[Title/Abstract])) OR ("transcatheter aortic valve replacement"[Title/Abstract])) OR ("heart surgery"[Title/Abstract])) OR ("cardiac surgery"[Title/Abstract])) OR ("cardiopulmonary bypass"[Title/Abstract])) OR ("valve surgery"[Title/Abstract])) OR ("coronary artery bypass surgery"[Title/Abstract]))) OR ((cardi*[Title/Abstract] OR heart[Title/Abstract] OR coronary[Title/Abstract] OR valv*[Title/Abstract] OR myocard*[Title/Abstract] OR thora*[ Title/Abstract]) AND (surg*[Title/Abstract] OR proced*[Title/Abstract] OR operat*[Title/Abstract] OR bypass[Title/Abstract]))) AND (("Delirium"[Mesh]) OR (((((((((((((delirium[Title/Abstract]) OR (agitation[Title/Abstract])) OR (confusion[Title/Abstract])) OR ("emergence delirium"[Title/Abstract])) OR ("postoperative delirium"[Title/Abstract])) OR ("postsurgical delirium"[Title/Abstract])) OR ("postoperative cognitive dysfunction"[Title/Abstract])) OR ("intensive care psychosis"[Title/Abstract])) OR ("drug induced psychosis"[Title/Abstract])) OR ("acute confusion"[Title/Abstract])) OR ("organic brain syndrome"[Title/Abstract])) OR ("acute encephalopathy"[Title/Abstract])) OR ("cognitive dysfunction"[Title/Abstract])))) AND (((((("Child"[Mesh]) OR ("Child, Preschool"[Mesh])) OR ("Adolescent"[Mesh])) OR ("Infant, Newborn"[Mesh])) OR ("Pediatrics"[Mesh])) OR ((((((((((((((((((((((child[Title/Abstract]) OR (pediatrics[Title/Abstract])) OR (children[Title/Abstract])) OR ("preschool child"[Title/Abstract])) OR ("children, preschool"[Title/Abstract])) OR ("preschool children"[Title/Abstract])) OR (toddler[Title/Abstract])) OR (adolescent*[Title/Abstract])) OR (adolescence[Title/Abstract])) OR (teen*[Title/Abstract])) OR (teenager*[Title/Abstract])) OR (youth*[Title/Abstract])) OR ("female adolescent*"[Title/Abstract])) OR ("male adolescent*"[Title/Abstract])) OR ("infant*, newborn"[Title/Abstract])) OR ("newborn infant*"[Title/Abstract])) OR (newborn*[Title/Abstract])) OR (neonate*[Title/Abstract])) OR (infant[Title/Abstract])) OR (baby[Title/Abstract])) OR (pediatric*[Title/Abstract])) OR (neonatology[Title/Abstract])))

**Web of science**

#1 TS=(“Cardiac Surgical Procedures”) OR TS=(“Thoracic Surgical Procedures”) OR TS=(“Thoracic Surgery”) OR TS=(“cardiac surgical procedure”) OR TS=(“thoracic surgical procedure”) OR TS=(“heart surgical procedure”) OR TS=(“heart surgical procedures”) OR TS=(“cardiac valve annuloplasty”) OR TS=(“mitral valve annuloplasty”) OR TS=(“heart massage”) OR TS=(“heart transplantation”) OR TS=(“heart-lung transplantation”) OR TS=(“heart valve prosthesis implantation”) OR TS=(“transcatheter aortic valve replacement”) OR TS=(“heart surgery”) OR TS=(“cardiac surgery”) OR TS=(“cardiopulmonary bypass”) OR TS=(“valve surgery”) OR TS=(“coronary artery bypass surgery”)

#2 TS=(cardi* ) OR TS=(heart) OR TS=(coronary) OR TS=(valv*) OR TS=(myocard*) OR TS=(thora*)

#3 TS=(surg* ) OR TS=(proced*) OR TS=(operat* ) OR TS=(bypass)

#4 #2 AND #3

#5 #1 OR #4

#6 TS=(delirium) OR TS=(agitation) OR TS=(confusion) OR TS=(“emergence delirium”) OR TS=(“postoperative delirium”) OR TS=(“postsurgical delirium”) OR TS=(“postoperative cognitive dysfunction”) OR TS=(“intensive care psychosis”) OR TS=(“drug induced psychosis”) OR TS=(“acute confusion”) OR TS=(“organic brain syndrome”) OR TS=(“acute encephalopathy”) OR TS=(“cognitive dysfunction”)

#7 TS=(child) OR TS=(pediatric*) OR TS=(children) OR TS=(“preschool child”) OR TS=(“children, preschool”) OR TS=(“preschool children”) OR TS=(toddler) OR TS=(adolescent*) OR TS=(adolescence) OR TS=(teen*) OR TS=(teenager*) OR TS=(youth*) OR TS=(“female adolescent*”) OR TS=(“male adolescent*”) OR TS=(“infant*, newborn”) OR TS=(“newborn infant*”) OR TS=(newborn*) OR TS=(neonate*) OR TS=(infant) OR TS=(baby) OR TS=(neonatology)

#8 #5 AND #6 AND #7

**Cochrane**

#1 MeSH descriptor: [Cardiac Surgical Procedures] explode all trees

#2 MeSH descriptor: [Thoracic Surgical Procedures] explode all trees

#3 MeSH descriptor: [Thoracic Surgical Procedures] explode all trees

#4 (“cardiac surgical procedures” OR “cardiac surgical procedure” OR “thoracic surgical procedures” OR “thoracic surgical procedure” OR “thoracic surgery” OR “heart surgical procedure” OR “heart surgical procedures” OR “cardiac valve annuloplasty” OR “mitral valve annuloplasty” OR “heart massage” OR “heart transplantation” OR “heart-lung transplantation” OR “heart valve prosthesis implantation” OR “transcatheter aortic valve replacement” OR “heart surgery” OR “cardiac surgery” OR “cardiopulmonary bypass” OR “valve surgery” OR “coronary artery bypass surgery”):ti,ab,kw

#5 #1 OR #2 OR #3 OR #4

#6 cardi* OR heart OR coronary OR valv* OR myocard* OR thora*

#7 surg* OR proced* OR operat* OR bypass

#8 #6 AND #7

#9 #5 OR #8

#10 MeSH descriptor: [Delirium] explode all trees

#11 (delirium OR agitation OR confusion OR “emergence delirium” OR “postoperative delirium” OR “postsurgical delirium” OR “postoperative cognitive dysfunction” OR “intensive care psychosis” OR “drug induced psychosis” OR “acute confusion” OR “organic brain syndrome” OR “acute encephalopathy” OR “cognitive dysfunction”):ti,ab,kw

#12 #10 OR #11

#13 MeSH descriptor: [Child] explode all trees

#14 MeSH descriptor: [Child, Preschool] explode all trees

#15 MeSH descriptor: [Infant, Newborn] explode all trees

#16 MeSH descriptor: [Adolescent] explode all trees

#17 MeSH descriptor: [Pediatrics] explode all trees

#18 (child OR pediatric* OR children OR “preschool child” OR “children, preschool” OR “preschool children” OR toddler OR adolescent* OR adolescence OR teen* OR teenager* OR youth* OR “female adolescent” OR “male adolescent” OR “infant, newborn” OR “newborn infant” OR newborn* OR neonate* OR infant OR baby OR neonatology):ti,ab,kw

#19 #13 OR #14 OR #15 OR #16 OR #17 OR #18

#20 #9 AND #12 AND #19

**Embase**

#1 'thorax surgery'/exp

#2 'heart surgery'/exp

#3 'cardiac surgical procedures':ti,ab,kw OR 'cardiac surgical procedure':ti,ab,kw OR 'thoracic surgical procedures':ti,ab,kw OR 'thoracic surgical procedure':ti,ab,kw OR 'thoracic surgery':ti,ab,kw OR 'heart surgical procedure':ti,ab,kw OR 'heart surgical procedures':ti,ab,kw OR 'cardiac valve annuloplasty':ti,ab,kw OR 'mitral valve annuloplasty':ti,ab,kw OR 'heart massage':ti,ab,kw OR 'heart transplantation':ti,ab,kw OR 'heart-lung transplantation':ti,ab,kw OR 'heart valve prosthesis implantation':ti,ab,kw OR 'transcatheter aortic valve replacement':ti,ab,kw OR 'heart surgery':ti,ab,kw OR 'cardiac surgery':ti,ab,kw OR 'cardiopulmonary bypass':ti,ab,kw OR 'valve surgery':ti,ab,kw OR 'coronary artery bypass surgery':ti,ab,kw

#4 #1 OR #2 OR #3

#5 cardi*:ti,ab,kw OR heart:ti,ab,kw OR coronary:ti,ab,kw OR valv*:ti,ab,kw OR myocard*:ti,ab,kw OR thora*:ti,ab,kw

#6 surg*:ti,ab,kw OR proced*:ti,ab,kw OR operat*:ti,ab,kw OR bypass:ti,ab,kw

#7 #5 AND #6

#8 #4 OR #7

#9 'delirium'/exp

#10 delirium:ti,ab,kw OR agitation:ti,ab,kw OR confusion:ti,ab,kw OR 'emergence delirium':ti,ab,kw OR 'postoperative delirium':ti,ab,kw OR 'postsurgical delirium':ti,ab,kw OR 'postoperative cognitive dysfunction':ti,ab,kw OR 'intensive care psychosis':ti,ab,kw OR 'drug induced psychosis':ti,ab,kw OR 'acute confusion':ti,ab,kw OR 'organic brain syndrome':ti,ab,kw OR 'acute encephalopathy':ti,ab,kw OR 'cognitive dysfunction':ti,ab,kw

#11 #9 OR #10

#12 'child'/exp

#13 'preschool child'/exp

#14 'adolescent'/exp

#15 'newborn'/exp

#16 'pediatrics'/exp

#17 child:ti,ab,kw OR pediatric*:ti,ab,kw OR children:ti,ab,kw OR 'preschool child':ti,ab,kw OR 'children, preschool':ti,ab,kw OR 'preschool children':ti,ab,kw OR toddler:ti,ab,kw OR adolescent*:ti,ab,kw OR adolescence:ti,ab,kw OR teen*:ti,ab,kw OR teenager*:ti,ab,kw OR youth*:ti,ab,kw OR 'female adolescent':ti,ab,kw OR 'male adolescent':ti,ab,kw OR 'infant, newborn':ti,ab,kw OR 'newborn infant':ti,ab,kw OR newborn*:ti,ab,kw OR neonate*:ti,ab,kw OR infant:ti,ab,kw OR baby:ti,ab,kw OR neonatology:ti,ab,kw

#18 #12 OR #13 OR #14 OR #15 OR #16 OR #17

#19 #8 AND #11 AND #18

**Scopus**

( ( TITLE-ABS-KEY ( "cardiac surgical procedures" OR "cardiac surgical procedure" OR "thoracic surgical procedures" OR "thoracic surgical procedure" OR "thoracic surgery" OR "heart surgical procedure" OR "heart surgical procedures" OR "cardiac valve annuloplasty" OR "mitral valve annuloplasty" OR "heart massage" OR "heart transplantation" OR "heart-lung transplantation" OR "heart valve prosthesis implantation" OR "transcatheter aortic valve replacement" OR "heart surgery" OR "cardiac surgery" OR "cardiopulmonary bypass" OR "valve surgery" OR "coronary artery bypass surgery" ) ) OR ( ( TITLE-ABS-KEY ( cardi* OR heart OR coronary OR valv* OR myocard* OR thora* ) ) AND ( TITLE-ABS-KEY ( surg* OR proced* OR operat* OR bypass ) ) ) ) AND ( TITLE-ABS-KEY ( delirium OR agitation OR confusion OR "emergence delirium" OR "postoperative delirium" OR "postsurgical delirium" OR "postoperative cognitive dysfunction" OR "intensive care psychosis" OR "drug induced psychosis" OR "acute confusion" OR "organic brain syndrome" OR "acute encephalopathy" OR "cognitive dysfunction" ) ) AND ( TITLE-ABS-KEY ( child OR pediatric* OR children OR "preschool child" OR "children, preschool" OR "preschool children" OR toddler OR adolescent* OR adolescence OR teen* OR teenager* OR youth* OR "female adolescent" OR "male adolescent" OR "infant, newborn" OR "newborn infant" OR newborn* OR neonate* OR infant OR baby OR neonatology ) )

**Sinomed**

("谵妄"[全部字段:智能] OR "急性脑综合征"[全部字段:智能] OR "谵妄发生"[全部字段:智能] OR "谵妄症状"[全部字段:智能] OR "谵妄状态"[全部字段:智能] OR "急性谵妄"[全部字段:智能] OR "精神错乱"[全部字段:智能] OR "急性精神错乱"[全部字段:智能] OR "认知功能障碍"[全部字段:智能]) AND ("心脏手术"[全部字段:智能] OR "心脏外科手术"[全部字段:智能] OR "心脏手术后"[全部字段:智能] OR "心脏移植"[全部字段:智能] OR "心肺移植"[全部字段:智能] OR "瓣膜手术"[全部字段:智能] OR "二尖瓣环成形术"[全部字段:智能] OR "搭桥手术"[全部字段:智能] ) AND ("儿童"[全部字段:智能] OR "儿科"[全部字段:智能] OR "学龄前儿童"[全部字段:智能] OR "学龄期儿童"[全部字段:智能] OR "青年"[全部字段:智能] OR "青少年"[全部字段:智能] OR "青少年儿童"[全部字段:智能] OR "新生儿"[全部字段:智能] OR "早产儿"[全部字段:智能])

**CNKI**

(主题:心脏手术 + 心脏外科手术 + 心脏手术后 + 手术 + 手术后 + 心脏移植+ 心肺移植 + 瓣膜手术+ 二尖瓣环成形术 + 搭桥手术(精确)) AND (主题: 谵妄 + 急性脑综合征 + 谵妄发生 + 谵妄症状 + 谵妄状态 + 急性谵妄 + 谵妄评估 + 精神错乱 + 急性精神错乱 + 认知功能障碍(精确)) AND (主题: 儿童 + 儿科 + 学龄前儿童 + 学龄期儿童 + 青年 + 青少年 + 青少年儿童 + 新生儿 + 早产儿(精确))

**Wanfang**

((主题=(心脏手术 or 心脏外科手术 or 心脏手术后 or 心脏移植 or 心肺移植 or 瓣膜手术 or 二尖瓣环成形术 or 搭桥手术)) AND 主题=(谵妄 or 急性脑综合征 or 谵妄发生 or 谵妄症状 or 谵妄状态 or 急性谵妄 or 谵妄评估 or 精神错乱 or 急性精神错乱 or 认知功能障碍)) AND 主题=(儿童 or 儿科 or 学龄前儿童 or 学龄期儿童 or 青年 or 青少年 or 青少年儿童 or 新生儿 or 早产儿)

**Supplemental Digital: Fig.S1-S3**


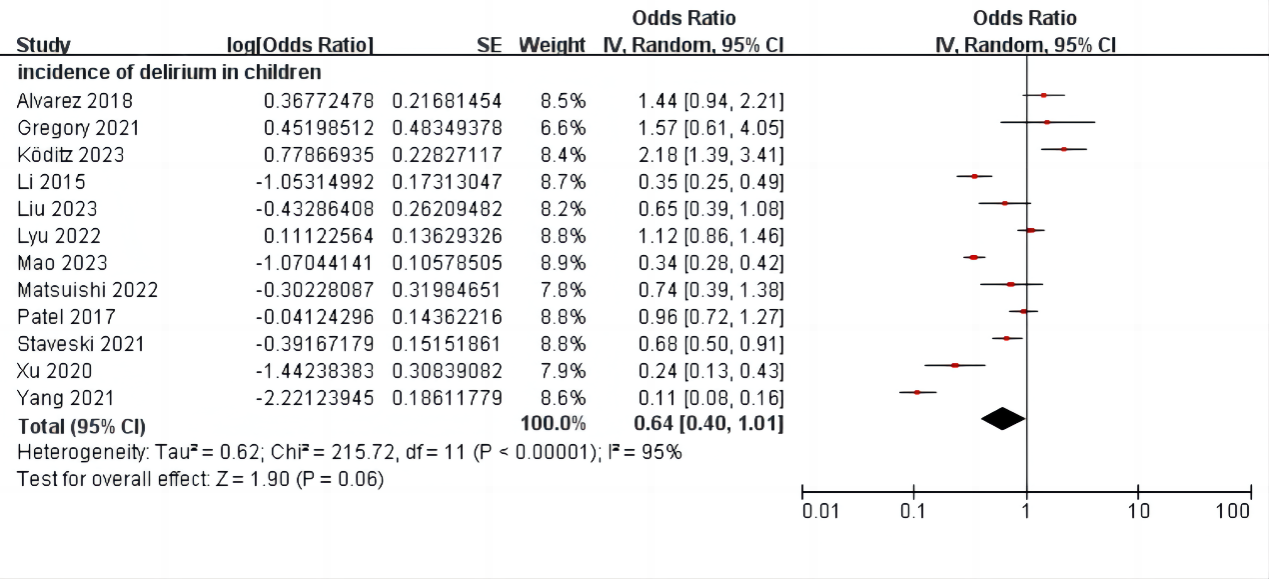


Fig. S1 Meta-integration of the incidence of delirium in children after cardiac surgery


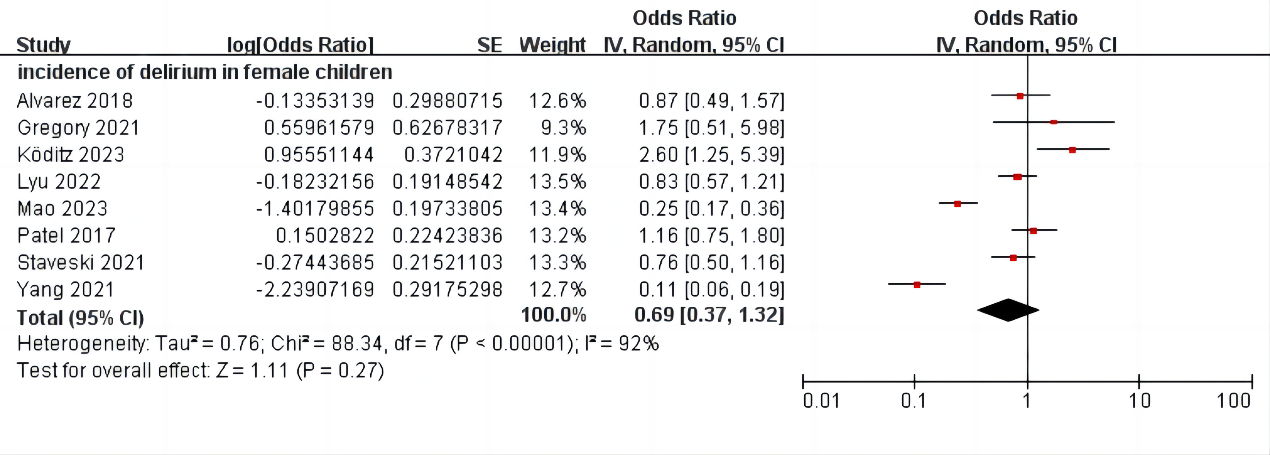


Fig. S2 Meta-integration of the incidence of delirium in female children after cardiac surgery


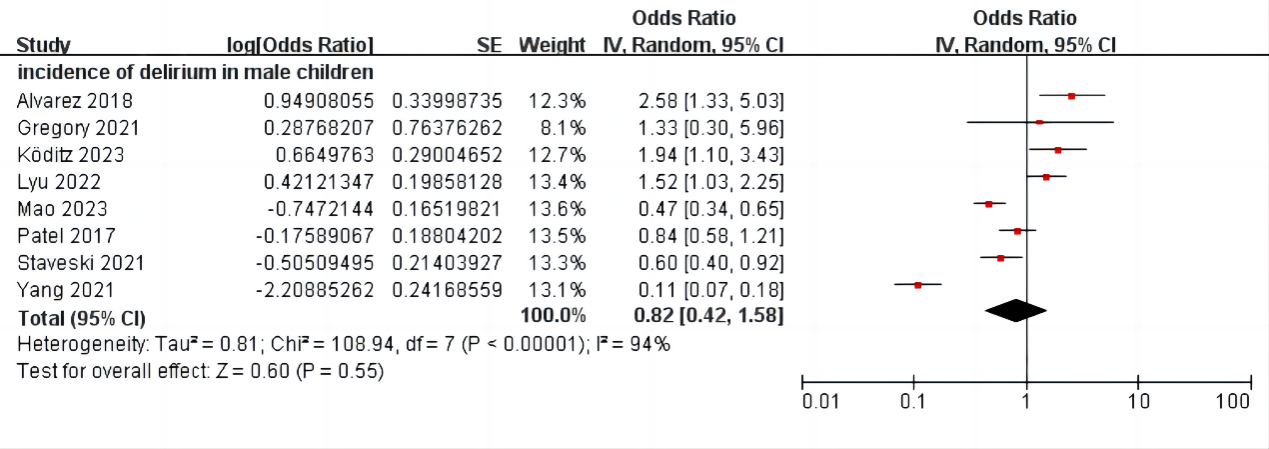


Fig. S3 Meta-integration of the incidence of delirium in male children after cardiac surgery


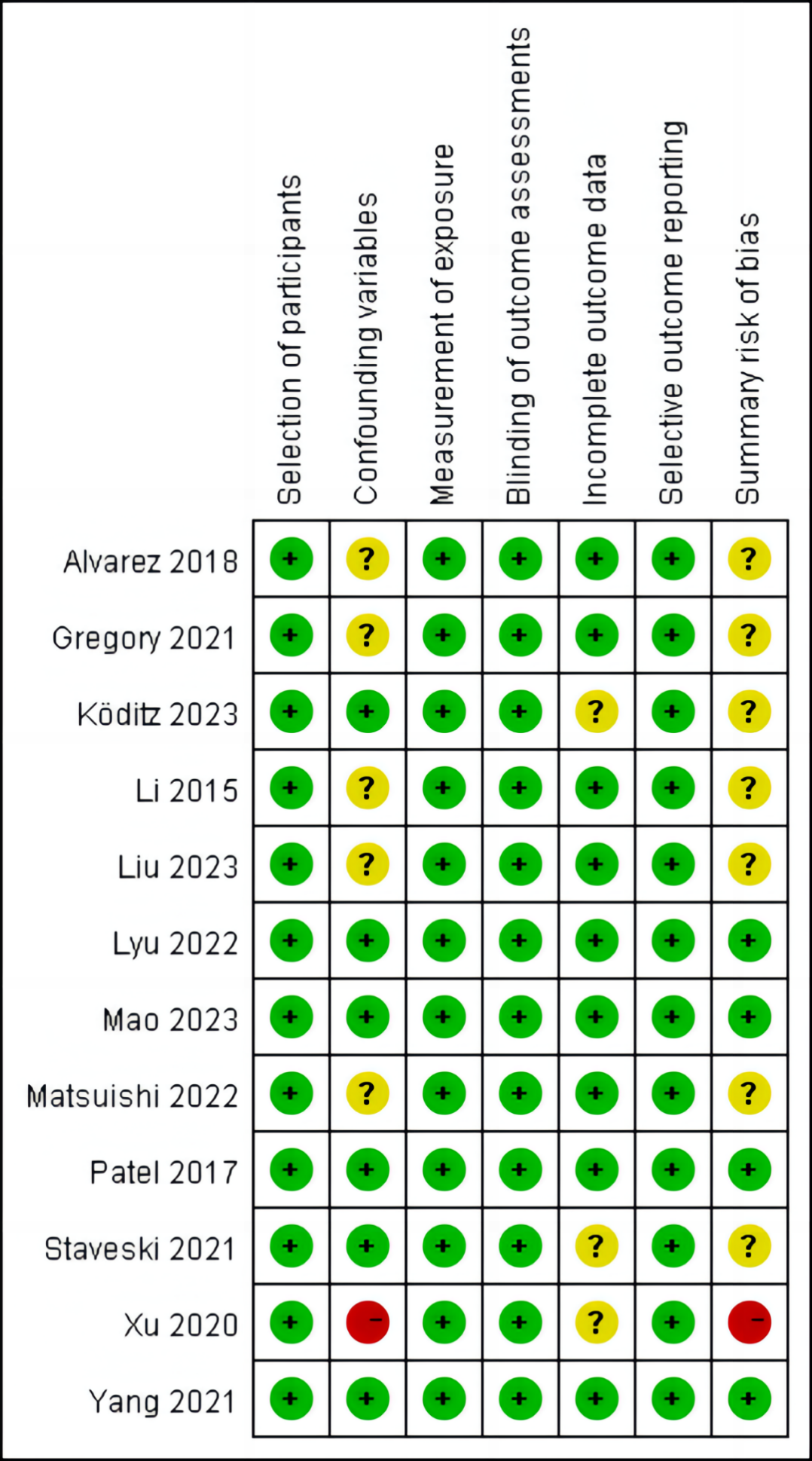


Fig. S4 Quality evaluation of each study
